# Intra‐ and inter‐fractional liver and lung tumor motions treated with SBRT under active breathing control

**DOI:** 10.1002/acm2.12220

**Published:** 2017-11-20

**Authors:** Lan Lu, Claudiu Diaconu, Toufik Djemil, Gregory MM Videtic, May Abdel‐Wahab, Naichang Yu, John Greskovich, Kevin L Stephans, Ping Xia

**Affiliations:** ^1^ Department of Radiation Oncology Taussig Cancer Center Cleveland Clinic Cleveland OH USA

**Keywords:** ABC, kV‐CBCTs, liver, lung, SBRT

## Abstract

**Purpose:**

To assess intra‐ and inter‐fractional motions of liver and lung tumors using active breathing control (ABC).

**Methods and Materials:**

Nineteen patients with liver cancer and 15 patients with lung cancer treated with stereotactic body radiotherapy (SBRT) were included in this retrospective study. All patients received a series of three CTs at simulation to test breath‐hold reproducibility. The centroids of the whole livers and of the lung tumors from the three CTs were compared to assess intra‐fraction variability. For 15 patients (8 liver, 7 lung), ABC‐gated kilovoltage cone‐beam CTs (kV‐CBCTs) were acquired prior to each treatment, and the centroids of the whole livers and of the lung tumors were also compared to those in the planning CTs to assess inter‐fraction variability.

**Results:**

Liver intra‐fractional systematic/random errors were 0.75/0.39 mm, 1.36/0.97 mm, and 1.55/1.41 mm at medial‐lateral (ML), anterior‐posterior (AP), and superior‐inferior (SI) directions, respectively. Lung intra‐fractional systematic/random errors were 0.71/0.54 mm (ML), 1.45/1.10 mm (AP), and 3.95/1.93 mm (SI), respectively. Substantial intra‐fraction motions (>3 mm) were observed in 26.3% of liver cancer patients and in 46.7% of lung cancer patients. For both liver and lung tumors, most inter‐fractional systematic and random errors were larger than the corresponding intra‐fractional errors. However, these inter‐fractional errors were mostly corrected by the treatment team prior to each treatment based on kV CBCT‐guided soft tissue alignment, thereby eliminating their effects on the treatment planning margins.

**Conclusions:**

Intra‐fractional motion is the key to determine the planning margins since inter‐fractional motion can be compensated based on daily gated soft tissue imaging guidance of CBCT. Patient‐specific treatment planning margins instead of recipe‐based margins were suggested, which can benefit mostly for the patients with small intra‐fractional motions.

## 1 INTRODUCTION

The success of stereotactic body radiation therapy (SBRT) relies heavily on the precise localization and immobilization of targeted tumors during the treatment to ensure accurate treatment delivery. Because of considerable organ motion, liver and lung tumors are especially challenging to immobilize. To manage the uncertainty, several techniques such as abdominal compression,^1^ deep inspiration voluntary breath‐hold (DIBH),^2^, ^3^ respiratory gating,^4^ four‐dimensional computed tomography,^5^, ^6^, ^7^ real‐time tumor tracking,^8^ and active breathing control^9^, ^10^, ^11^, ^12^ have been implemented. The choice of the motion management method would affect the intra‐ and inter‐fraction planning margins. Active breathing control (ABC) involves suspending the patient's breathing at a predetermined breathing phase.^12^, ^13^ Various studies reported the results of intra‐ and inter‐fractional organ motion using the ABC technique for liver and lung cancer.^14^, ^15^, ^16^, ^17^, ^18^ These reported intra‐ and inter‐fractional organ motions have large variations, heavily depending on the method of data collection and analysis. Several of these early studies used 2D radiography, which may not provide adequate 3D information to assess tumors in liver or lung accurately. Some of them reported inter‐fractional displacement comprising both setup error and organ motion. Applying these inconsistent data to clinical practice is challenging. To date, kilovoltage cone‐beam computed tomography (kV‐CBCT) has been widely used to provide 3D information for soft tissues which can significantly improve the precision of radiotherapy.^5^, ^19^ The purpose of this study was to assess the intra‐fractional motion (the variation in motion within one fraction) and inter‐fractional motion (the variation between different fractions) of liver and lung tumors using the ABC technique and kV‐CBCT, and investigate the roles of the intra‐ and inter‐fractional motions in the treatment planning margins. In the current study, with initially correcting patient positioning errors in six dimensions using a pair of stereoscopic KV images, inter‐fraction tumor position changes were measured and further corrected using kV‐CBCT‐guided soft tissue alignment. With this procedure, we were able to separate the patient positioning errors from inter‐fractional organ motion and correct them separately.

## 2 PATIENTS AND METHODS

### 2.A Patients

Thirty‐four patients treated with ABC‐guided SBRT between May 2010 and June 2012 (19 patients with liver cancer or liver metastases and 15 patients with lung cancer) were randomly selected from an IRB‐approved registry. All patients underwent three consecutive CTs at simulation with each acquisition on a separate ABC‐gated breath‐hold to verify the reproducibility of breath‐hold. As the function of the gated kilovoltage cone‐beam CT was available later in our department, only 15 patients (8 liver and 7 lung) had acquired gated CBCT prior to each treatment fraction. At our institution, patients receiving liver SBRT were frequently treated with ABC and three fractions with a typical total prescription dose of 37.5 Gy. Patients receiving lung SBRT were mostly treated with abdominal compression if tumor motion can be restricted to <5–10 mm. Patients whose breathing motion cannot be controlled by the compression were instead treated with ABC, which were included in the current observed cohort. The prescription dose for these patients was typically 50 Gy in five consecutive fractions. The time from the simulation to the first day of treatment for all SBRT patients was approximately 10 business days.

### 2.B Simulation session

Patients were positioned supine with arms above the head and immobilized with BlueBAG™ BodyFIX^®^ cushions (Elekta, Stockholm, Sweden). Using the Elekta^®^ Active Breathing Coordinator device (Elekta), the airflow was blocked with a nose clip while patients underwent breath‐hold, and airflow flowed through while opening the balloon valve or releasing mouthpiece when patients resume the normal breathing. Before CT acquisition, patients underwent a training session to reproducibly hold their breath at 75%–80% of maximal inspiratory volume for at least 15–20 s. Without repositioning the patient between scans, three consecutive CTs (3 mm slice thickness) were obtained. These CTs for lung patients were acquired without contrast. Triphasic CTs were acquired for liver patients, including precontrast (first CT), followed by arterial (second CT) and venous (third CT) phase scans on subsequent breath‐holds after the administration of IV contrast.

### 2.C Treatment session

During treatment, patients were positioned with the same immobilization device used in simulation and then aligned with infrared markers equipped in the ExacTrac system (Brainlab, Feldkirchen, Germany). ABC training was briefly repeated at the first treatment session. Subsequently, a set of stereographic X‐ray images were taken with the patient holding breath under ABC. The stereographic X‐ray images were then aligned to the spine to correct for setup errors in six dimensions using a robotic couch. After setup correction, a respiratory gated kV‐CBCT scan was acquired while the patient held breath under ABC with the same breath‐hold threshold determined during simulation. A further correction was performed based on soft tissue alignment of liver or lung tumor by registering CBCT with planning CT, which can provide online correction of the inter‐fractional organ motions. Figure 1 illustrates the schematic diagram of the workflow about the imaging acquisitions and corresponding patient alignments. A whole CBCT acquisition took 60 s for a full 360 degree rotation, thereby three to four breath‐holds were needed for a single‐gated CBCT scan. We used the “stop‐and‐go” CBCT technique, as previously described.^20^ The CBCT slice thickness was 2.5 mm. The beam‐on time and number of breath‐hold for a patient during treatment depended on the prescription dose, duration of the breath‐hold, number of beams, total number of MUs, and the maximum dose rate of the beam.

**Figure 1:**
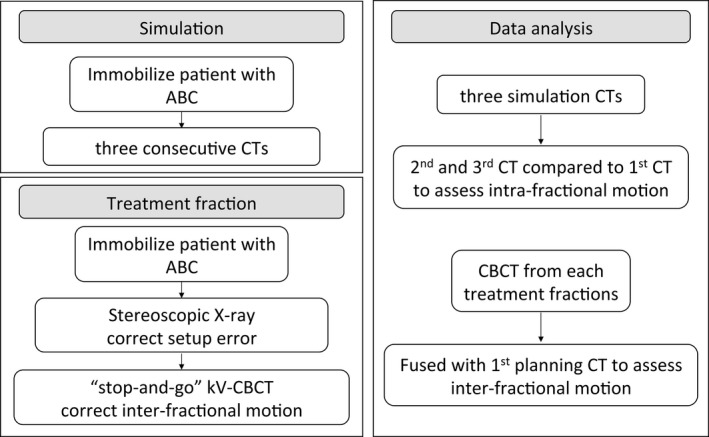
Schematic diagram of image acquisition and data analysis.

### 2.D Data analysis

As shown in Fig. 1, three consecutive CTs obtained during the simulation were used to assess intra‐fraction variability by comparing the second and third CT scans to the first one. To assess inter‐fraction variability, each CBCT from the treatment sessions was compared to the planning CT. For each patient with lung cancer, the gross tumor volume (GTV) was visualized and contoured on three simulation CTs and on three CBCTs. For each patient with liver cancer, because the liver GTVs were difficult to delineate in some CTs and most CBCTs, the centroids of the whole livers were used as surrogates for the centroids of liver tumors. Therefore, the whole livers were contoured on three simulation CTs and three CBCTs from different treatment days.

After rigid image registration to the vertebral body to remove potential residual position errors or patient movement, the centroid shifts of the lung tumor and liver were measured between the planning CT and other simulation CTs to obtain intra‐fraction organ motion and between the planning CT and CBCTs to obtain inter‐fraction organ motion at medial‐lateral (ML), anterior‐posterior (AP), and superior‐inferior (SI) directions, respectively. Subsequently, these shifts were used to obtain the overall group mean error (∆M, which is the mean of all patients' mean shifts), the systematic error (Σ, which is the standard deviation around group mean error), and the random error (*σ*, which is defined as the root mean square of the patients' standard deviations), as previously described.^21^

## 3 RESULTS

The distribution of individual patient intra‐ and inter‐fractional motions of liver and lung is presented in Fig. 2. The liver intra‐fractional motion was measured in 19 liver cancer patients [Fig. 2(a)]. Five (26.3%) patients exhibited intra‐fractional displacements of the liver >3 mm. Among them, two (10%) patients had intra‐fractional motion larger than 4 mm in the SI direction only. No displacement >5 mm was observed in any direction for these patients. The lung intra‐fractional motion was measured in 15 patients [Fig. 2(b)]. Seven (46.7%) patients exhibited intra‐fractional displacements of the tumor >3 mm. Among them, four (26.7%) had intra‐fractional motion between 5 and 10 mm, and one (6.7%) had motion >10 mm, all of which was in the SI direction. For both liver and lung, inter‐fractional motion exhibited larger variation than intra‐fractional motion and most large displacements were found in the SI direction. The liver inter‐fractional motion was assessed in eight patients [Fig. 2(c)] and lung inter‐fractional motion was measured in seven patients [Fig. 2(d)] based on registration of the CBCT and planing CT. Seven (87.5%) patients exhibited liver inter‐fractional displacements >3 mm. Among them, one (12.5%) patient had inter‐fractional motion between 5 and 10 mm, and one (12.5%) had inter‐fractional motion >10 mm. Similarly, six (85.7%) patients with lung cancer exhibited lung inter‐fractional displacements >3 mm, three (43%) of which had inter‐fractional motion between 5 and 10 mm. No patient had motion >10 mm.

**Figure 2:**
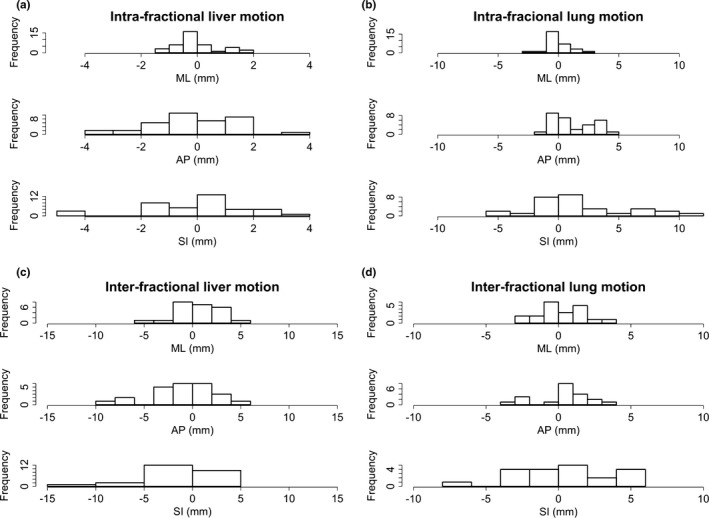
Intra‐ and inter‐fractional liver and lung motion at medial‐lateral, anterior‐posterior, and superior‐inferior direction, respectively.

Figure 3 summarizes the group of intra‐ and inter‐fractional motions for both liver and lung tumors, respectively, at ML, AP, and SI directions using box plots. From Fig. 3, the range of liver inter‐fractional displacements was much larger than the liver intra‐fractional displacements, while the range of lung inter‐fractional displacement was comparable to lung intra‐fractional displacements at AP and SI directions. The mean absolute intra‐ and inter‐fractional displacements in the liver at ML, AP, and SI directions were 0.59 mm #bib1.16 mm, 1.33 mm, and 1.78 mm, 2.64 mm, 2.97 mm, respectively. The mean absolute intra‐ and inter‐fractional displacements in the lung tumor at ML, AP, and SI directions were 0.64 mm, 1.55 mm, 2.93 mm, and 1.14 mm, 1.42 mm, 2.79 mm. It is worthy of note that although the inter‐fractional displacements were substantial, these displacements were mostly corrected prior treatment based on CBCT‐guided soft tissue alignment.

**Figure 3:**
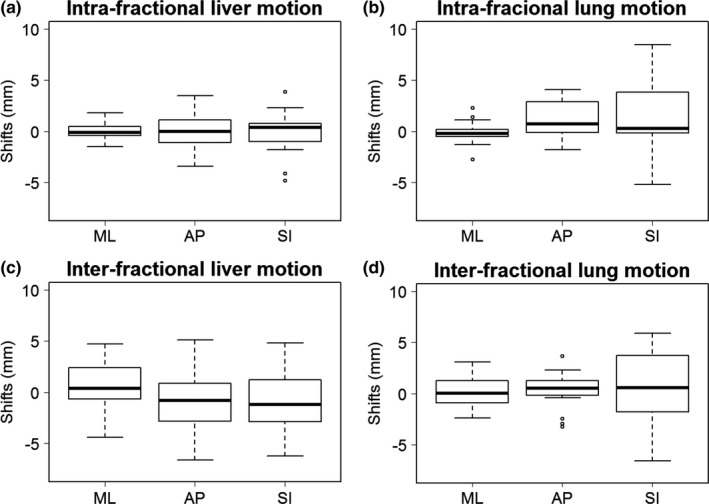
Box plot of intra‐ and inter‐fractional motions for livers and lung tumors at medial‐lateral, anterior‐posterior, and superior‐inferior direction, respectively. Thick lines represent the median value, box represents 25th and 75th percentile of the shifts, the dots or outer lines represent the range of the shifts.

Table 1 shows the calculated intra‐ and inter‐fraction reproducibility errors in the ML, AP, and SI directions. For both liver and lung cancer patients, the group mean errors (ΔM) of intra‐ and inter‐fraction were less than 2 mm in all directions with a maximum value of 1.81 mm for lung intra‐fraction motion in the SI direction. For liver cancer patients, most of the inter‐fractional systematic (Σ) and random (σ) errors were greater than those of intra‐fraction errors, which could be due to the lower soft tissue contrast of the liver in CBCT images. For patients with lung cancer, the measured intra‐ and inter‐fraction reproducibility errors were comparable. The greatest error was the systematic error in the SI direction.

**Table 1:** Group mean error (ΔM), systematic error (Σ), and random error (*σ*) for intra‐ and inter‐fractional reproducibility

	ΔM (mm)			∑ (mm)			*σ* (mm)		
	ML	AP	SI	ML	AP	SI	ML	AP	SI
Liver CTs	0.03	−0.18	−0.02	0.75	1.36	1.55	0.39	0.97	1.41
Liver CBCTs	0.67	−0.93	−1.33	1.77	3.20	3.87	1.62	1.77	1.86
Lung CTs	−0.07	1.26	1.81	0.71	1.45	3.95	0.54	1.10	1.93
Lung CBCTs	0.16	0.29	0.70	1.37	1.80	2.93	0.73	0.87	2.54

ML, medial‐lateral; AP, anterior‐posterior; SI, superior‐inferior.

## 4 DISCUSSION

In this study, we measured intra‐ and inter‐fractional motion of liver and lung tumors for patients treated with SBRT under ABC. With CBCT corrections, inter‐fractional motion was compensated; therefore, intra‐fractional motion is the key to design the planning margins. Our study showed that the majority (74%) of liver cancer patients and half (56%) of the lung cancer patients had small (<3 mm) intra‐fractional motions. Acquiring three consecutive CTs during simulation, we can obtain patient‐specific intra‐fraction organ motion, which can reduce unnecessary toxicities for the patients who were compliant with the ABC and had small intra‐fraction organ motion.

Because of considerable organ motion, liver and lung tumors are especially challenging to localize during SBRT treatment. Active breathing control (ABC) is one of the methods to control the tumor motion^12^, ^13^, ^15^ and has been widely used to reduce the breathing motion for treating both lung and liver tumors.^4^, ^11^, ^13^, ^14^, ^15^, ^20^ Early reproducibility studies showed that lung tumor displacements of 0–5 mm still occurred despite using the ABC technique.^14^, ^16^ More recently, a study from Brock et al^22^ showed that intra‐fraction absolute displacements were around 1.7 mm (ML) #bib1.5 mm (AP), and 1.7 mm (SI), similar to our results: 0.64 mm (ML), 1.55 mm (AP), and 2.93 mm (SI); however, inter‐fraction displacements (3.6 mm (ML), 3.5 mm (AP), and 5.1 mm (SI)) were much greater than what we observed (1.14 mm (ML), 1.42 mm (AP), and 2.79 mm (SI)). A possible explanation is that the inter‐fraction displacements in the reference^22^ were determined from CT obtained weeks apart throughout a 6.5‐week treatment course. Using five continuous days of CT to evaluate the ability of ABC to immobilize peripheral lung tumors, Cheung et al measured that the displacements of GTV centers were 0.3 mm (±1.8 mm) #bib1.2 mm (±2.3 mm), and 1.1 mm (±3.5 mm) in ML, AP, and SI directions, respectively.^14^ Those mean displacements and standard deviation are much more consistent to that we reported with the displacements of 0.16 mm (±1.46 mm) #bib0.29 mm (±1.79 mm), and 0.74 mm (±3.42 mm) in ML, AP, and SI directions, respectively.

Several other studies also eliminated the initial setup errors with initial bony structure alignment. For instance, after removing the initial setup errors by aligning to bony anatomy in the mediolateral and anteroposterior directions and aligning to the diaphragm in the superoinferior direction, Hawkins et al^20^ analyzed the inter‐fraction liver motion using the orthogonal MV portal images and orthogonal KV planner images acquired after repositioning. Because the initial setup correction in the superoinferior direction was aligned to the diaphragm, the reported inter‐fraction liver motion in the superoinferior direction was 1.6 mm (absolute max = 5.0 mm) which was smaller than that of the present study (mean = 1.33 mm, absolute max = 12.8 mm) and the other study.^23^ Zhong et al analyzed the inter‐ and intra‐fraction liver motion based on three sets of non‐gated CBCTs for each patient, including precorrection CBCT, pretreatment CBCT, and post‐treatment CBCT.^23^ Because the setup errors were not explicitly separated from the liver motion in the precorrection CBCT scans, the reported inter‐fractional systematic/random errors from their study which were 3.18/3.03 mm #bib3.05/3.62 mm, and 6.80/6.78 mm in ML, AP, and SI directions, respectively, are larger than the present study (Table 1). Zhong et al applied the same method to analyze the inter‐ and intra‐fractional motion in lung tumors,^24^ and their reported systematic/random errors of tumor reproducibility were 4.5/2.6 mm (ML) #bib4.0/3.6 mm (AP), and 5.1/4.8 mm (SI), which were also larger than that of the present study (Table 1).

Intra‐ and inter‐fractional organ motions for liver and lung tumors have large variations, depending on the method of data collection and analysis. Careful consideration of details of the methods should be undertaken when applying these data to clinical practice. If the inter‐fraction motion is compensated, the margins for organ motion can be further reduced to less than 5 mm for patients with liver cancer and to less than 10 mm for patients with lung cancer. It should be noted that lung cancer patients in our study are those whose tumor motion could not be controlled by abdominal compression. This selection bias might contribute to the relatively larger inter‐ and intra‐fraction tumor motion reported by our study.

In this study, we did not use the van Herk formula^21^ to calculate the population‐based planning margins, which was derived to guarantee that 90% of patients in the population receive a minimum cumulative CTV dose of at least 95% of the prescribed dose for a conventional fractionation. First, directly applying this formula to SBRT treatment with three to five fractions may be questionable. Second, to guarantee 90% of patient population receiving a minimum cumulative dose of at least 95% of the prescription dose, this formula may heavily influence patients on both ends of the organ motion spectrum. For example, if directly using the formula, the predicted intra‐fraction organ motion margins would be 2.1 mm (ML) #bib4.1 mm (AP), and 4.9 mm (SI) for liver and 2.2 mm (ML), 4.4 mm (AP), and 11.0 mm (SI) for lung. However, the majority (73%) of liver patients and half (54%) of the lung patients exhibited intra‐fraction displacements less than 3 mm, but only one patient experienced lung tumor motion greater than 10 mm. It may not be judicious to use recipe‐based margins for those patients with smaller (<3 mm) intra‐fractional motion. In addition, since KV‐cone‐beam CT has been widely available clinically, compensating the inter‐fraction motion is clinically possible and essential for SBRT patients with liver and lung cancer. To further reduce intra‐fraction planning margins, considering patient‐specific intra‐fractional organ motions, instead of recipe‐based margins, may be necessary.

As discussed above, the direct use of the planning margin for organ motion requires caution as these may depend on the details of patient immobilization methods and imaging guidance procedures. One limitation of the current study is our limited ability to visualize liver tumors on CTs and, in particular, on CBCTs. As such, most studies used various surrogates for liver tumor motion, including whole‐liver contours,^20^, ^23^ diaphragm dome position relative to the vertebra,^15^, ^25^ and hepatic microcoils.^15^ Another limitation is we did not further acquire a postcorrection CBCT to verify if our soft tissue alignment had fully corrected the inter‐fractional motion as Zhong et al did.^23^, ^24^ Their studies showed that residual errors were still present in the verified CBCT after initial correction. The cause of these residual errors was very likely the patient movement. However, in the current study, we used orthogonal X‐ray to confirm the consistence of bony structures prior treatment to verify the stability of patient's position. Since the patients recruited in the current project did not have implanted markers, we used intra‐fractional motion measured at simulation as surrogate of the motion during the treatment. In future studies, we will recruit patients with implanted markers and acquire triggered images during treatment to verify the consistency of intra‐fractional motions between simulation and during the treatment. In addition, to investigate the impact of motion of internal moving organ on the clinical end points, the follow‐up study will also focus on the comparison of the dosimetric results with and without taking account of these motions.

## 5 CONCLUSION

The reproducibility of ABC becomes of great importance for patients with liver and lung cancer treated with SBRT. Using daily KV‐CBCT, soft tissue image guidance to correct for the inter‐fraction motion is essential for this group of patients. With CBCT corrections, substantial inter‐fractional motion can be compensated; therefore, intra‐fractional motion is the key to determine the planning margins. Our study indicated that intra‐fractional motions were small (<3 mm) for the majority (74%) of liver cancer patients and half (56%) of the lung cancer patients. With three consecutive CTs acquired during simulation, we can obtain patient‐specific intra‐fraction organ motion, which can be of benefit for patients compliant with the use of ABC and who had small intra‐fraction organ motion, thus reducing planning margins and reducing unnecessary toxicities.

## CONFLICT OF INTEREST

The contents are solely the responsibility of the authors and do not necessarily represent the official views of Johns Hopkins University or the National Cancer Institute.
